# Neural markers of a greater female responsiveness to social stimuli

**DOI:** 10.1186/1471-2202-9-56

**Published:** 2008-06-30

**Authors:** Alice M Proverbio, Alberto Zani, Roberta Adorni

**Affiliations:** 1 Dept. of Psychology, University of Milano-Bicocca, Italy; 2 Inst. of Molecular Bioimaging and Physiology, CNR, Milano-Segrate, Italy

## Abstract

**Background:**

There is fMRI evidence that women are neurally predisposed to process infant laughter and crying. Other findings show that women might be more empathic and sensitive than men to emotional facial expressions. However, no gender difference in the brain responses to persons and unanimated scenes has hitherto been demonstrated.

**Results:**

Twenty-four men and women viewed 220 images portraying persons or landscapes and ERPs were recorded from 128 sites. In women, but not in men, the N2 component (210–270) was much larger to persons than to scenes. swLORETA showed significant bilateral activation of FG (BA19/37) in both genders when viewing persons as opposed to scenes. Only women showed a source of activity in the STG and in the right MOG (extra-striate body area, EBA), and only men in the left parahippocampal area (PPA).

**Conclusion:**

A significant gender difference was found in activation of the left and right STG (BA22) and the cingulate cortex for the subtractive condition women minus men, thus indicating that women might have a greater preference or interest for social stimuli (faces and persons).

## Background

That women are more interested than men in conspecifics, as opposed for example to machinery, is a common prejudice not really substantiated by scientific evidence. It is true, however, that female children across various human cultures are prone to spend more time with their younger siblings, or their simulacra (baby dolls), than are their male counterparts. It is quite difficult to determine whether this socially-oriented behaviour is entirely due to cultural factors (such as the style of upbringing) or to a biological difference dependent on genetic factors. Indeed, as shown in psychosocial studies [1-3] the "affective education" received by females differs from a very young age from that given to males. Consequently, women develop a more pronounced tendency to empathy and towards understanding the verbal and non-verbal signals inherent in the behaviour of others. Other studies [4] have tended to stress the genetic/biological nature of female preference for social stimuli. Indeed, in a interesting study on toy preference in nonhuman primates (Cercopithecus aethiops sabaeus), it was found that the percentage contact time with toys that are preferred by boys (a car and a ball) was greater in male than female vervets, whereas the percentage contact time with toys that are typically preferred by girls (dolls) was greater in female than male vervets. These data hint that preferences for sexually differentiated objects arose early in human evolution, prior to the emergence of a distinct hominid lineage. This study is especially important since, unlike humans, monkeys are not subject to the specific social and cognitive influences proposed to explain human gender differences in toy preference.

Whatever the cause, little neuroscientific evidence of such preference of the female brain for social stimuli has been reported, in contrast to the large body of behavioural evidence showing that females have greater social and affective competence. For example, a substantial literature has accumulated indicating that women are better than men at decoding facial expressions of emotion [5-10]. Various studies have demonstrated differences between the ways in which men and women perceive, process, express and experience emotions. Generally speaking, women seem more able, as well as more inclined, to express their own emotions to conspecifics [11]. Furthermore, they show greater ease in decoding non-verbal indicators connected to the expression of emotions.

An interesting fMRI study [12] provided the first evidence of a difference in brain response to social stimuli in women and men listening to infants crying and laughing: women but not men showed deactivation in the anterior cingulate cortex in response to both infant crying and laughter. This gender effect per se was interpreted as a preference of female individuals for the specific sensory signals (infant vocalizations). In a more recent study [13], infant laughter and crying elicited stronger activation in the amygdala and anterior cingulate cortex (ACC) of women than men. According to the authors, this indicated that women are neurally predisposed to respond to preverbal infant vocalizations. However, these two findings are somewhat contradictory, but both point to the involvement of ACC in gender differences in brain responses to social stimuli.

Some fMRI [14] and bioelectrical [15] evidence indicates that women might be more empathic than men when viewing suffering people. For example, Singer and colleagues engaged male and female volunteers in an economic game, in which two confederates played fairly or unfairly. They found that while women activated empathy- and pain-related brain areas (fronto-insular and anterior cingulate cortices) every time either a fair or an unfair person received pain, these empathy-related responses were significantly lower in males when they observed an unfair person receiving pain. In a recent ERP study [16] it was found that visual evoked responses to infant faces were larger and earlier in women than men, and again this was interpreted as a sign of the female brain's greater interest in/preference for this class of biologically relevant stimuli (infant faces).

**Figure 1:**
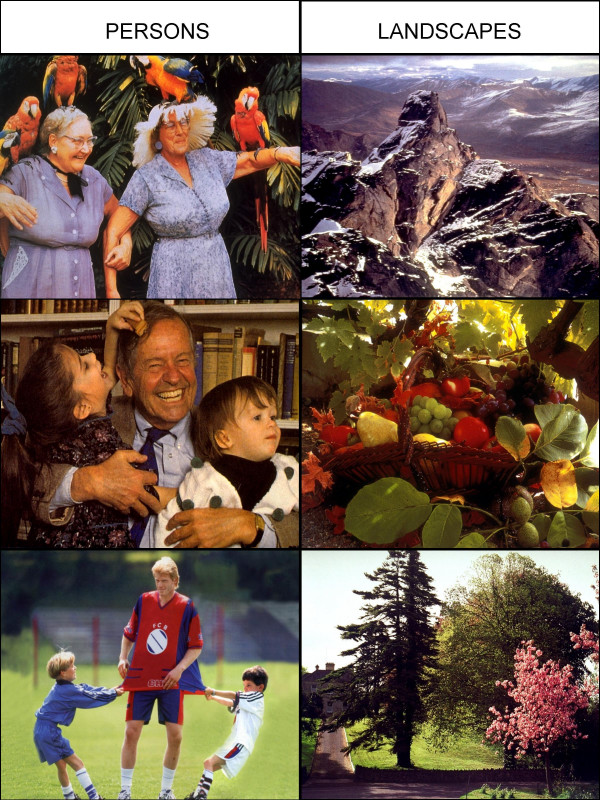
**Stimuli**. Exemplars of IAPS stimuli showing social scenes (persons) or scenes without visible persons (landscapes).

Otherwise, the neurobiological bases of a gender difference in brain responsiveness to social stimuli remain unexplored. The goal of the present study was to shed some light on this matter by measuring brain bio-electrical activities in men and women during perception of positive social scenes vs. landscapes (some examples are shown in Fig. 1).

The valence of affective pictures taken from the International Affective Picture System (IAPS) [17] has been shown to modulate the amplitude of multiple ERP components: going from the early extra-striate P1 response [18], found to be larger to negative than positive IAPS stimuli, through the P2 component [19], found to be larger to negative than positive pictures, to the late positive complex [20,21], consistently larger to positive and negative than neutral scenes. Overall, it seems that positively-valenced pictures are processed later than aversive/fearful pictures. For example, in an ERP study of face processing it was found that, while occipito/temporal N170 was affected by the emotional content of negative pictures, differentiating strongly negative (pain) from weakly negative (discomfort) expressions, the fine processing of positive emotions occurred later (namely at pre-frontal areas at about 245 ms of latency [6]). In the present study, all pictures were roughly equivalent in terms of valence (positive) and arousal; they only differed in terms of content (humans vs. scenes). Therefore, we hypothesized that any gender difference in the amplitude of ERP components as a function of stimulus content was to be ascribed to the presence/absence of social information (conspecifics) rather than to the affective component of the visual information.

## Results

**Figure 2:**
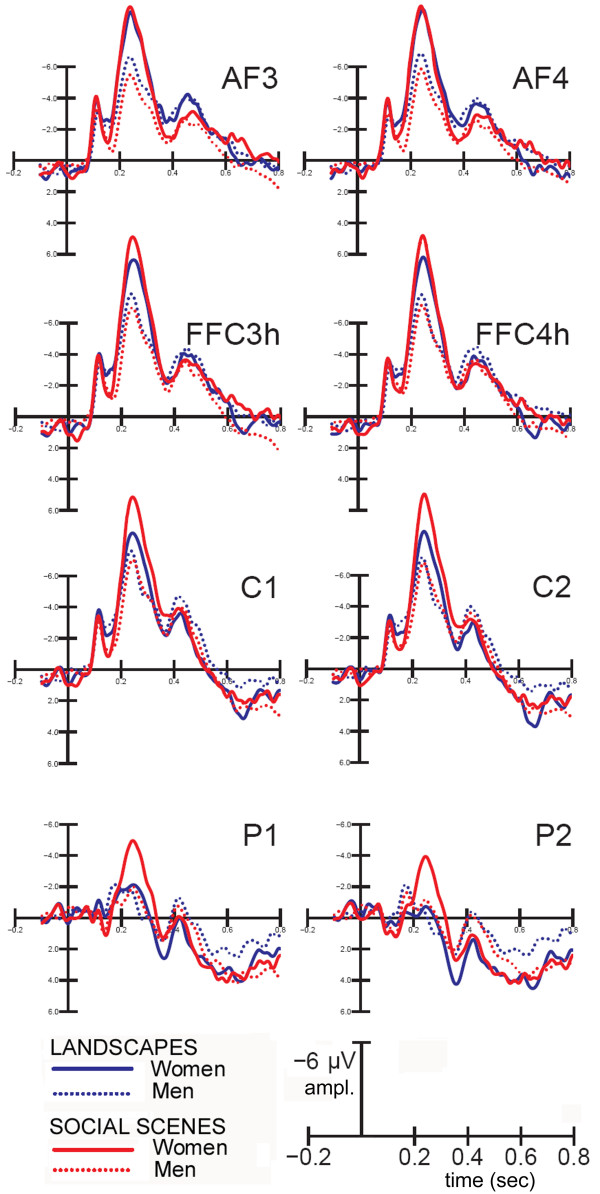
**ERPs recorded in women and men in response to persons and scenes**. Grand-average ERPs recorded from the left and right anterior frontal, fronto-central, central and parietal sites according to viewer gender and stimulus content.

Fig. 2 shows the grand-average ERPs recorded as a function of gender and scene type, evidencing important differences at frontal and parietal sites at the level of the anterior N2 component. Analysis of variance showed the significance of electrode (F3,66 = 81.95; p < 0.0000) and electrode × hemisphere (F3,66 = 14.03; p < 0.00000) factors, indicating a larger N2 response at fronto-central sites, bilaterally, and a strong left hemispheric asymmetry at parietal sites.

The interaction of scene type × gender (F1,22 = 10.5, p < 0.0004) and relative post-hoc comparisons showed significantly (p < 0.02) larger N2 responses to social scenes (-8.85 μV, SE = 1.381) than landscapes (-7.21 μV, SE = 1.148) in women, whereas men showed a tendency (p < 0.08) toward the opposite preference (landscapes = -5.62 μV, SE = 1.148; social scenes = -4.88 μV, SE = 1.381).

The ANOVA also showed that scene type × electrode (F3,66 = 31; p < 0.0000) and scene type × electrode × hemisphere (F3,66 = 9.6; p < 0.00002) were significant. Post-hoc comparisons indicated that the parietal N2 was left-sided and was larger to social scenes than landscapes, whereas the bilateral anterior frontal N2 was larger to landscapes than social scenes.

**Figure 3:**
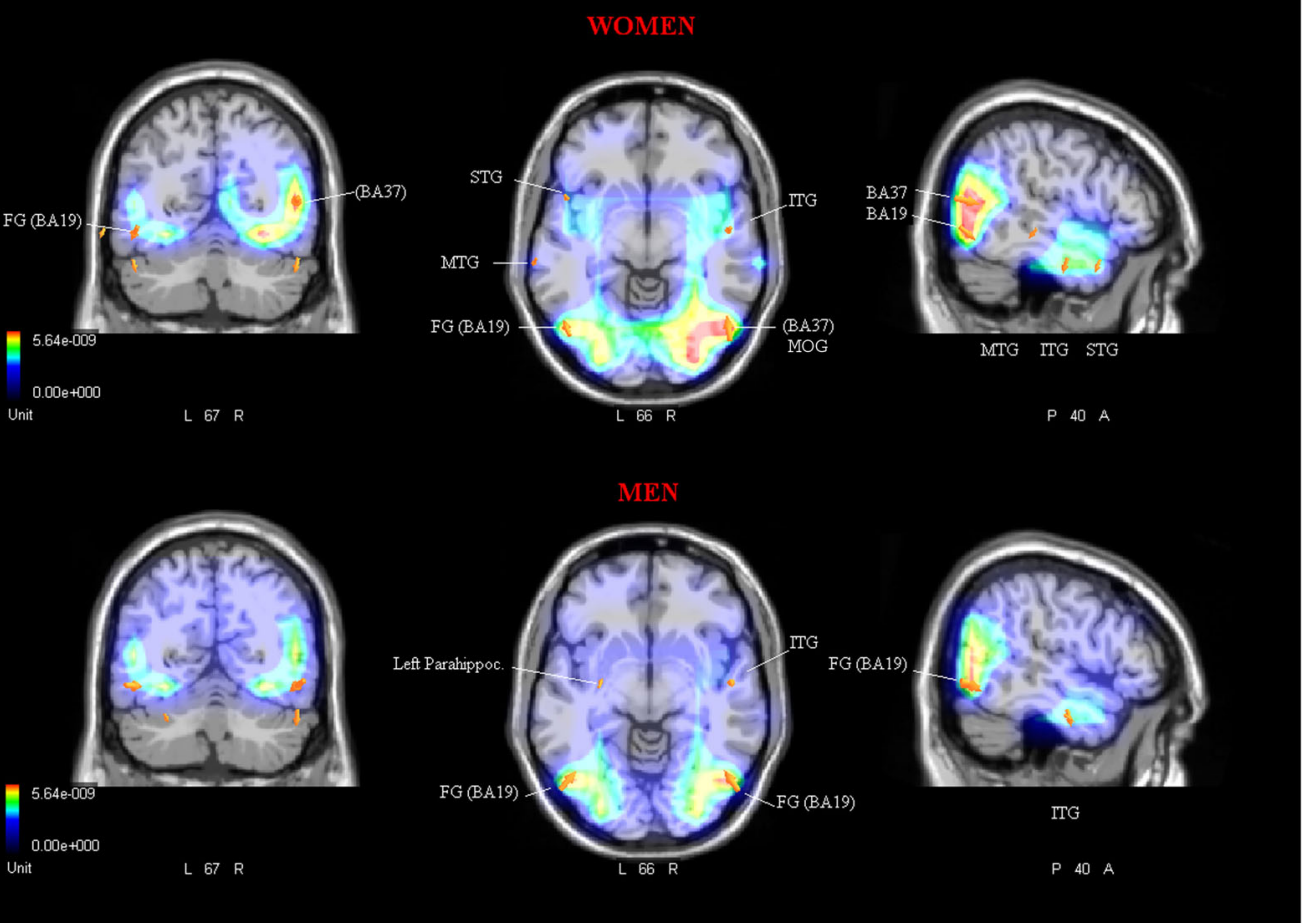
**Persons – scenes**. Coronal, axial and sagittal views of significant intracranial sources of activation for the contrast Persons-Scenes in the latency range 210–270 ms corresponding to the peak of anterior N2, separately for women and men (N = 24).

Two swLORETA source reconstructions were performed (shown in Fig. 3), separately for men and women, on the difference waves obtained by subtracting ERPs to landscapes from ERPs to social scenes in the time window 210–270 ms, corresponding to the N2 latency range. This contrast allowed us to observe which brain regions were involved in the response to human bodies, faces and social interactions, as opposed to unanimated scenes. Table 1 provides a list of significantly active sources explaining the different surface voltages and the Tailarach coordinates of their corresponding neural generators. For both genders, significant sources of activation were located in the left and right fusiform gyrus (FG, BA19). Furthermore, both women and men exhibited activation of the right inferior temporal gyrus (ITG, BA20). Only women showed a source of activity in the right middle occipital gyrus (MOG), and in the superior temporal gyrus (STG), and only men showed activation of the left parahippocampal gyrus.

**Table 1:** Persons – scenes.

**Magn (E-09)**	**T-x (mm)**	**T-y (mm)**	**T-z (mm)**	**Hem.**	**Area**
**Women**					

7.40	50.8	-68	4.7	RH	Occipital lobe, Middle Occipital Gyrus, BA 37
5.84	-48.5	-66.1	-10.9	LH	Temporal lobe, Fusiform gyrus, BA19
4.73	50.8	-8.7	-21.5	RH	Temporal lobe, Inferior temporal gyrus, BA20
3.76	-68.5	-25.5	-8.1	LH	Temporal lobe, Middle temporal gyrus, BA21
4.01	-48.5	8.2	-20	LH	Temporal lobe, Superior temporal gyrus, BA38

**Men**					

6.21	50.8	-66.1	-10.9	RH	Temporal lobe, Fusiform gyrus, BA19
5.89	-48.5	-66.1	-10.9	LH	Temporal lobe, Fusiform gyrus, BA19
4.25	50.8	-8.7	-21.5	RH	Temporal lobe, Inferior temporal gyrus, BA20
3.07	-28.5	-8.7	-21.5	LH	Parahiccocampal gyrus, Hippocampus

Tailarach coordinates corresponding to the intracranial generators explaining the difference voltages Persons – Scenes in the 210–270 ms time window, according to swLORETA (ASA) [49]; grid spacing = 10 mm.

**Figure 4:**
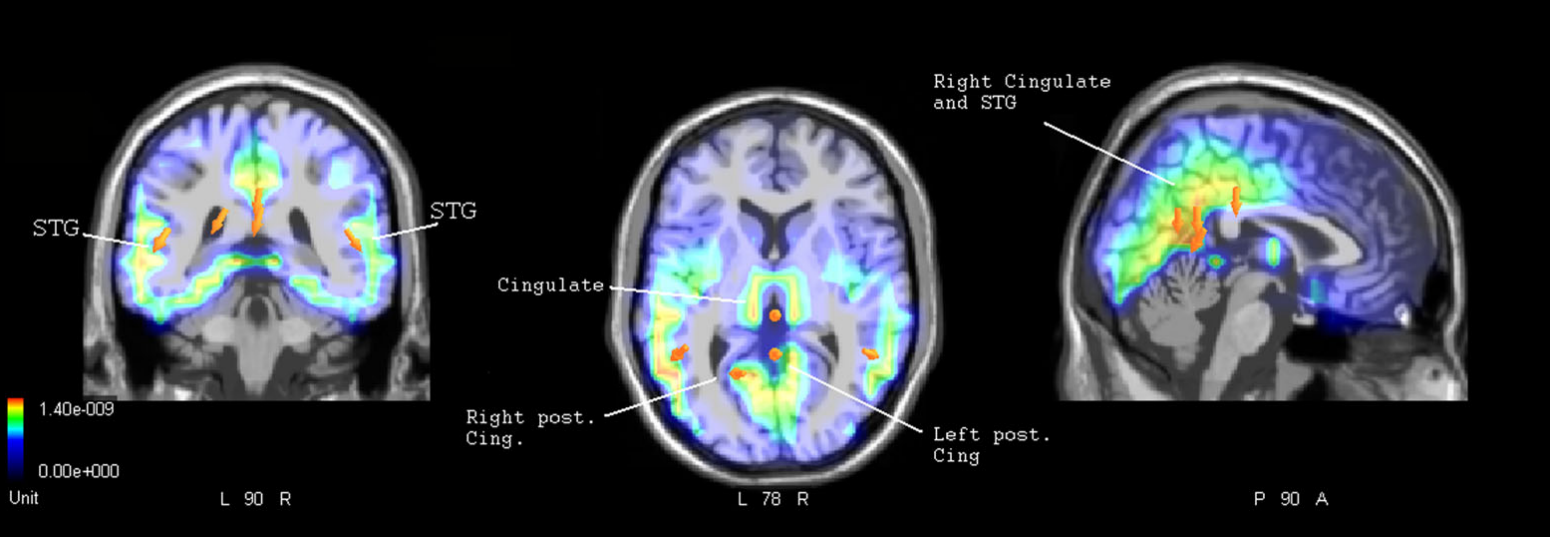
**Women – men**. Coronal, axial and sagittal views of significant intracranial sources of activation for the contrast Women-Men relative to the Persons-Scenes difference voltage computed in the latency range 220–260 ms corresponding to the peak of anterior N2.

To test for statistically significant gender differences in brain activation, a further swLORETA source reconstruction was performed (Fig. 4; relative active sources are listed in Table 2) on the difference wave obtained by subtracting ERPs to Persons-Scenes recorded in Men (N = 12) from ERPs to Persons-Scenes recorded in women (N = 12). This source reconstruction showed a gender difference in the activation of the left and right STG (BA22), of the left and right posterior cingulate and of the right cingulate cortex (CC), with significantly stronger activation of these regions in the female than the male brain in response to conspecifics rather than scenes.

**Table 2:** Women – men.

**Magn (E-09)**	**T-x (mm)**	**T-y (mm)**	**T-z (mm)**	**Hem.**	**Area**
**Women – Men**					

1.40	-48.5	-47.8	6.4	LH	Temporal lobe, Superior Temporal Gyrus, BA22
1.38	1.5	-48.7	15.3	RH	Posterior Cingulate, BA30
1.32	-18.5	-58.9	14.5	LH	Posterior Cingulate, BA20
1.32	1.5	-29.4	26	RH	Cingulate Gyrus, BA23
1.24	50.8	-47.8	6.4	RH	Temporal lobe, Superior Temporal Gyrus, BA22

Tailarach coordinates corresponding to the intracranial generators explaining the difference voltage obtained by subtracting ERPs to Persons-Scenes in men from ERPs to Persons-Scenes in women, according to swLORETA (ASA) performed for the 220–260 ms time window; grid spacing = 5 mm.

## Discussion

In this study all stimuli were positively-valenced. The mean valences for the two classes of stimuli were 7.01 for social scenes and 6.9 for landscapes, according to the data by Lang and colleagues [17]. The mean arousal levels were 4.88 for social stimuli and 4.24 for scenes. As a result, ERP components typically affected by affective picture processing ([18-23] did not differ, and especially did not differ between genders. N1 was also insensitive to scene content. Indeed it has been shown that positively-valenced pictures are processed later than aversive/fearful pictures. The N2 response was the only ERP component showing a significant and conspicuous gender effect in interaction with scene type.

The data showed a marked gender difference at about 201–270 ms in the bioelectrical response to social scenes Vs. landscapes with no visible persons, evidenced by a much larger parietal N2 component in response to conspecifics in female than in male viewers. LORETA source reconstruction evidenced a strong involvement of the left and especially the right FG of the temporal cortex (BA19/37), of the right MOG and of the right STG during processing of persons vs. scenes. The fusiform face area (FFA) has been described as a portion of the extra-striate cortex specialized for face perception; it responds markedly more strongly to faces than to other classes of objects e.g. hands, houses, scrambled faces [24]. Several TMS and fMRI studies [25-27] have also identified a region in the lateral occipito/temporal cortex (MOG) strongly involved in the visual perception of the human body (extra-striate body area, EBA) located near the so-called face visual area (FFA) [24].

In this context, a larger activation of FFA and EBA in women than men might indicate greater interest in or attention to this class of biologically relevant signals (human faces and bodies) in those individuals who are genetically determined to be the primary offspring caregivers. Since no affective behavioural response or attention allocation to scenes was required by the task, this gender difference might reflect a privileged automatic processing of visual images depicting conspecifics in the female brain. Naturally, since this is the first neural evidence of such a finding, further investigation with higher spatial resolution techniques will help to shed light on this matter. Indeed, it remains unclear whether the greater activation in response to conspecifics found in the right extra-striate cortex, or in the higher-order STG, of the female brain might be the result of visual learning/expertise or be modulated by neuro-hormonal factors. For example, the neuropeptide oxytocin is known to play an important role in affiliation behaviour such as pair-bonding and maternal care. Rather recently, oxytocin has been found to be a potent modulator in the processing of social stimuli, improving trust in social interactions [28], and even promoting the ability to infer the affective mental states of others from subtle facial cues [29].

On the other hand, the greater left parahippocampal gyrus activation in men than women might be due to their interest in the non-human components of scenarios surrounding the persons depicted. Indeed, it is known that a subregion of the parahippocampal cortex (the parahippocampal place area, PPA) plays an important role in encoding topographical scene stimuli such as images of landscapes, cityscapes or rooms (i.e. images of "places") [30,31].

As for the specific gender difference observed in the activation of the right STG and CC, it can be noted that both regions are thought to be involved in the perception of affective visual images [32], social cognition [33] and theory of mind such as understanding intentions [34]. In more detail, the STS is also involved in the processing of facial expressions of emotion, being particularly sensitive to the perception of social signals such as direction of gaze, speech-related lip movements and other changeable aspects of faces [35,36]. Furthermore, specific STS activation has been reported for perceiving fear in dynamic body expressions [37], processing of complex social signals such as facial expressions and body images [38], observation of actions and understanding [39]. Greater activation of this region in women than men might indicate greater interest in or allocation of attention to social aspects of visual information.

There is considerable evidence that the CC and ACC has a particular role in empathy [40-43]. In a recent fMRI study [44] simulating pain perception, adopting the perspective of the other (rather than self) was associated with a specific increase in the posterior cingulate/precuneus and the right temporo-parietal junction. Similarly, an fMRI study in which subjects viewed affective faces and either focused on their own emotional response to each face (self-task) or evaluated the emotional state expressed by the face (other-task) showed that the self- (relative to the other-) task caused differential activation of – among other areas – the posterior cingulate cortex (PCC)/precuneus, and the temporo-parietal junction bilaterally [45]. Our finding that the cingulate cortex (BA 20, 23,30) is more greatly activated in women than men in response to conspecifics might reflect the activation of neural circuits subserving human empathy.

One possible limitation of this study is that scenes were not actively attended to (attention was paid to abstract stimuli) and this might (or might not) have affected the gender difference. Further investigation is needed to reach a definitive conclusion.

## Methods

### Subjects

Twenty-four healthy right-handed Italian University students (12 males and 12 females) were recruited for this experiment. All were of middle-high socio-cultural status and their mean age was 22 years. All had normal or corrected-to-normal vision and reported no history of psychiatric diseases, neurological illness or drug abuse. Their handedness was assessed by the Italian version of Edinburgh Handedness Inventory [46], a laterality preference questionnaire reporting strong right-handedness and right ocular dominance in all participants. Experiments were conducted with the understanding and the written consent of each participant. The experimental protocol was approved by the ethical and research review board of the National Research Council in Milan.

### Stimuli and procedure

Two hundred and twenty positive IAPS slides were selected from the *International Affective Picture System *[17] (The list of all the slides used in this study is reported in the Appendix section). Half the pictures showed complex ecological scenes without visible persons (natural landscapes or attractive objects), the other half showed social scenes in which persons differing in age, gender and number were depicted. Erotic pictures were not included since strong gender differences in the physiological and cerebral response to nudes are known. Stimulus content was controlled as for number of persons depicted and gender of poser. Among the 110 social scenes, 48 depicted single individuals and 62 depicted two or more persons. Their genders were as follows: 63 slides showed 1 or more women; 68 slides showed 1 or more men; in 37 cases men and women were together; in 16 cases the gender was not clearly intelligible (e.g. skiers, cosmonauts, newborns). The social interactions depicted included people standing in a comfortable position, holding each other, smiling, playing, practicing sports, walking or running. To balance for motion content, humans were almost static in 59 of the pictures, while they were moving in 51.

The mean valences for the two classes of stimuli were 7.01 for social scenes and 6.9 for landscapes, according to the data by Lang and coworkers [17], which provide standardized values for the basic dimensions of emotion as rated by the Self-Assessment Manikin (SAM) on a scale from 1 to 9 [47]. The scores indicate that the pictures induced a positive emotional state in the viewer. The mean arousal levels were 4.88 for social stimuli and 4.24 for scenes. Stimuli were equiluminant across categories: Social stimuli = 41.28 cd/cm^2^; Landscapes = 39.98 cd/cm^2^). Stimuli were also balanced for perceptual complexity by choosing interesting scenarios rich in detail, but the overall spatial frequency content of each picture was not controlled for, leading to a possible limitation. Stimuli were randomly presented in the centre of the screen for about 1 s with an ISI of 2.8–3.2 s.

The 220 positive images were presented randomly mixed with 220 negative affective pictures. Stimuli were randomly presented in the centre of the screen for about 1 s with an ISI of 2.8–3.2 s.

Participants sat comfortably in a darkened, acoustically and electrically shielded cubicle and were instructed to fixate the centre of the screen and to avoid any eye or body movements during the recording session. The task consisted of responding to 72 target stimuli randomly mixed with the IAPS images, by pressing a button as accurately and quickly as possible with the index finger of the left or right hand. Abstract geometrical paintings all alike in structure but differing in colour were used as targets. The two hands were used alternately during the recording session. The response hand and order of sequences were counterbalanced across subjects.

### EEG recording and analysis

The EEG was continuously recorded from 128 sites at a sampling rate of 512 Hz. Horizontal and vertical eye movements were also recorded. Linked ears served as the reference lead. The EEG and electro-oculogram (EOG) were amplified with a half-amplitude band pass of 0.016–100 Hz. Electrode impedance was kept below 5 kΩ. EEG epochs were synchronized with the onset of stimulus presentation and analyzed by ANT-*EEProbe *software. Computerized artefact rejection was performed before averaging to discard epochs in which eye movements, blinks, excessive muscle potentials or amplifier blocking occurred. The artefact rejection criterion was a peak-to-peak amplitude exceeding 50 μV, and the rejection rate was ~5%. ERPs were averaged offline from -200 ms before to 1000 ms after stimulus onset.

The mean amplitude of the N2 component of the ERP was measured in the time window 210–270 ms at the left and right anterior frontal (AF3, AF4), central (C1, C2), fronto-central (FFC3h, FFC4h) and parietal (P1, P2) sites.

ERP data were subjected to multifactorial repeated-measures ANOVA. Factors were "scene type" (landscape, social scene), "electrode" (4 levels) and "hemisphere" (left, right). Post-hoc Tukey tests were used for multiple comparisons of means.

Topographical voltage maps of ERPs were made by plotting colour-coded isopotentials derived by interpolating voltage values between scalp electrodes at specific latencies. Low Resolution Electromagnetic Tomography (LORETA) [48] was performed on ERP difference waves at various time latencies with ASA4 software (ANT). LORETA, which is a discrete linear solution to the inverse EEG problem, corresponds to the 3D distribution of electric neuronal activity that has maximum similarity (i.e. maximum synchronization), in terms of orientation and strength, between neighbouring neuronal populations (represented by adjacent voxels). Source space properties were: grid spacing = 10 or 5 mm; Tikhonov regularization: estimated SNR = 3. In this study an improved version of Standardized Low-Resolution brain Electromagnetic Tomography (sLORETA) was used that incorporates a singular value decomposition-based lead field weighting: swLORETA [49].

## Conclusion

Our data indicate that the female human brain reacts strongly to the view of scenes involving humans rather than unanimated scenes.

The larger activation of the right extra-striate cortex (BA37) in women than men might indicate greater attention to this class of biologically relevant signals (conspecifics) in those individuals who are genetically determined to be the primary offspring caregivers. The stronger activation of affective brain areas (superior temporal gyrus and cingulate cortex) also suggests a possible gender difference in the empathic reaction to social scenes.

## Authors' contributions

AMP conceived and designed the study, accomplished most of the data analyses and wrote the manuscript. AZ and RA performed source reconstruction analysis. All authors read and approved the final version of the manuscript.

## Appendix

IAPS images:

Social stimuli, 1340, 1601, 2030, 2037, 2040, 2057, 2058, 2070, 2071, 2080, 2091, 2092, 2152, 2154, 2160, 2165, 2170, 2208, 2209, 2222, 2224, 2260, 2299, 2311, 2331, 2332, 2339, 2340, 2341, 2345, 2360, 2362, 2370, 2373, 2387, 2388, 2389, 2391, 2395, 2398, 2501, 2510, 2530, 2550, 2598, 2655, 2660, 2900.2, 4006, 4150, 4220, 4250, 4520, 4532, 4533, 4542, 4572, 4574, 4599, 4601, 4603, 4606, 4609, 4610, 4614, 4623, 4624, 4625, 4626, 4640, 4641, 4689, 4700, 5410, 5470, 5621, 5831, 5836, 7325, 8021, 8031, 8032, 8033, 8034, 8040, 8041, 8080, 8120, 8130, 8161, 8185, 8186, 8193, 8200, 8205, 8250, 8280, 8300, 8320, 8330, 8350, 8370, 8380, 8400, 8460, 8461, 8470, 8496, 8497, 8540.

Landscapes, 1333, 1419, 1440, 1441, 1450, 1460, 1463, 1500, 1510, 1540, 1560, 1590, 1600, 1602, 1603, 1604, 1610, 1620, 1640, 1650, 1660, 1661, 1670, 1675, 1710, 1720, 1721, 1722, 1740, 1750, 1810, 1811, 1812, 1900, 1910, 1920, 1942, 1947, 7057, 7192, 8500, 8501, 8510, 7330, 7430, 7450, 7220, 7230, 7260, 7280, 7282, 7286, 7291, 7320, 7472, 7480, 7481, 1731, 2791, 5000, 5001, 5010, 5020, 5030, 5200, 5201, 5220, 5260, 5300, 5450, 5480, 5551, 5593, 5594, 5600, 5611, 5631, 5660, 5700, 5711, 5720, 5731, 5750, 5760, 5779, 5780, 5781, 5800, 5811, 5814, 5820, 5849, 5870, 5890, 5891, 5910, 5982, 5991, 5994, 7039, 7242, 7490, 7495, 7501, 7508, 7510, 7545, 7580, 8162, 8170.
